# Identification of a prognostic model based on costimulatory molecule-related subtypes and characterization of tumor microenvironment infiltration in acute myeloid leukemia

**DOI:** 10.3389/fgene.2022.973319

**Published:** 2022-08-19

**Authors:** Yan Mao, Zhengyun Hu, Xuejiao Xu, Jinwen Xu, Chuyan Wu, Feng Jiang, Guoping Zhou

**Affiliations:** ^1^ Department of Pediatrics, The First Affiliated Hospital of Nanjing Medical University, Nanjing, China; ^2^ Department of Pediatrics, Shanghai Songjiang District Central Hospital, Shanghai, China; ^3^ Department of Pediatric Nephrology, Wuxi Children’s Hospital Affiliated to Nanjing Medical University, Wuxi, China; ^4^ Department of Rehabilitation Medicine, The First Affiliated Hospital of Nanjing Medical University, Nanjing, China; ^5^ Department of Neonatology, Obstetrics and Gynecology Hospital of Fudan University, Shanghai, China

**Keywords:** costimulatory molecule, tumor microenvironment, acute myeloid leukemia, overall survival, prognosis

## Abstract

Costimulatory molecules have been found to play significant roles in anti-tumor immune responses, and are deemed to serve as promising targets for adjunctive cancer immunotherapies. However, the roles of costimulatory molecule-related genes (CMRGs) in the tumor microenvironment (TME) of acute myeloid leukemia (AML) remain unclear. In this study, we described the CMRG alterations in the genetic and transcriptional fields in AML samples chosen from two datasets. We next evaluated their expression and identified two distinct costimulatory molecule subtypes, which showed that the alterations of CMRGs related to clinical features, immune cell infiltration, and prognosis of patients with AML. Then, a costimulatory molecule-based signature for predicting the overall survival of AML patients was constructed, and the predictive capability of the proposed signature was validated in AML patients. Moreover, the constructed costimulatory molecule risk model was significantly associated with chemotherapeutic drug sensitivity of AML patients. In addition, the identified genes in the proposed prognostic signature might play roles in pediatric AML. CMRGs were found to be potentially important in the AML through our comprehensive analysis. These findings may contribute to improving our understanding of CMRGs in patients with AML, as well as provide new opportunities to assess prognosis and develop more effective immunotherapies.

## 1 Introduction

Acute myeloid leukemia (AML) is one of the most prevalent hematological tumors. It is defined by the increase of undifferentiated myeloid progenitor cells in the hematopoietic system (Short et al., 2018). For decades, chemotherapy with or without transplantation has been the standard treatment for AML patients (Mazzarella et al., 2014; Bose et al., 2017; Jiang et al., 2021a). Despite advances in drug extraction, therapeutic care, and early detection, the overall long-term survival of AML patients remains dismal (Zeidan et al., 2019; Jiang et al., 2021b; Jiang et al., 2021c). Therefore, identifying novel and effective biomarkers, as well as prognostic risk models, become urgent.

The tumor microenvironment (TME) plays a crucial role in AML growth, development, and therapy (Jiang et al., 2022). In TME, T cells often aid in the differentiation of cancerous cells from healthy cells. Before launching the second assault, the naïve T cells must be activated by two signals, a specific antigen that can be recognized by receptors on the T cells and nonspecific costimulatory molecule signals (Bluestone, 1995). By changing the latter, cancer cells could prevent the recognition and escape from the attack (Sanmamed and Chen, 2018). Apart from the checkpoint pathway belonging to the B7-CD28 family (Janakiram et al., 2015; Zhang et al., 2018), costimulatory molecular signals also contain molecules from the tumor necrosis factor (TNF) family (Ward-Kavanagh et al., 2016). These costimulatory molecule-related genes (CMRGs) are possible targets for the creation of new immune therapies, and they may be good supplements to current methods (Croft et al., 2013; Schildberg et al., 2016). However, the majority CMRGs’ expression and their clinical implications in AML remain unknown.

This work systematically assessed the expression patterns of CMRGs and obtained a complete picture of the intra-tumoral immunological landscape *via* using CIBERSORT and ESTIMATE algorithms. First of all, expression levels of CMRGs were used to divide a total of 242 AML patients into two clustered costimulatory molecule subgroups. AML patients were then classified into CMRG-related gene subtypes according to those chosen differentially expressed genes between two costimulatory molecule subtypes. In addition, we constructed a signature that accurately predicted the clinical outcomes of AML patients and characterized the AML immune landscape. Our work, in a nutshell, systematically describes the landscape of costimulatory molecules and highlights their potential applications clinically, so aiding the creation of a rationale to guide AML patient care and treatment.

## 2 Methods and materials

### 2.1 Acquisition of data

Gene expression and relevant clinicopathological information on AML were from databases named The Cancer Genome Atlas (TCGA) (https://portal.gdc.cancer.gov/) and the Gene Expression Omnibus (GEO) (https://www.ncbi.nlm.nih.gov/geo/). One GEO AML cohort named GSE10358 and the TCGA-AML cohort were obtained for further relative analyses. The fragments per kilobase million (FPKM) values from the TCGA-AML set were converted to transcripts per kilobase million (TPM) and were then assumed to be the same as those from microarrays (Conesa et al., 2016; Zhao et al., 2021). The two chosen AML cohorts were combined. We removed data from individuals for whom we had no information on their overall survival; hence, 242 AML patients were included in our analysis. The clinical factors shared by the two AML groups were gender, age, duration of follow-up, and survival status. To confirm our proposed prognostic risk model, we also gathered expression data of pediatric AML samples from the Therapeutically Applicable Research to Generate Effective Treatments (TARGET) database (https://ocg.cancer.gov/programs/target) as an external validation cohort. Whole blood cohorts from GTEx downloaded from the UCSC Xena database (https://xenabrowser.net/datapages/) served as control samples for the analyses on the TCGA-AML and TARGET-AML samples.

### 2.2 Clustering analyses of costimulatory molecule-related genes

A total of 60 CMRGs were retrieved from previous publications (Zhang et al., 2020), as shown in Supplementary Table S1. We used the package named “ConsensusClusterPlus” in R software to perform consensus clustering analysis. The criteria were as follows: Firstly, the cumulative distribution function (CDF) curve should increase gradually and smoothly. Then, the size of all groups was large enough. Finally, the intra-group correlation should increase after clustering, while the inter-group correlation decreased. Also, gene set variation analysis (GSVA) was carried out using the hallmark gene set (c2. cp.kegg.v7.2) to investigate differences in chosen CMRGs in the biological processes.

### 2.3 Relationship between costimulatory molecule subtypes and clinical features of acute myeloid leukemia patients

We evaluated the relationships between clustered costimulatory molecule subtypes, clinical features, and outcomes. The characteristics mainly included gender and age. In addition, Kaplan-Meier curves, which were created using the “survival” and “survminer” packages, were used to evaluate the differences in overall survival across various costimulatory molecule subtypes.

### 2.4 Correlations of costimulatory molecule subtypes with tumor microenvironment in patients with acute myeloid leukemia

We assessed the immune, estimate and stromal scores of each AML sample in this study by using the ESTIMATE algorithm. Using the CIBERSORT algorithm, the fractions of 22 immune cell subgroups of each AML sample were estimated (Newman et al., 2015). The immune cell infiltration in the TME of AML was also identified by using an algorithm named single-sample gene set enrichment analysis (ssGSEA) (Rooney et al., 2015).

### 2.5 Identification of differentially expressed genes and annotation of their functions in detail

Differentially expressed genes between the two different costimulatory molecule subtypes were identified in R software. To explore the probable activities of costimulatory molecule profile-related differentially expressed genes and determine associated functions, we used “clusterprofiler” package to carry out functional enrichment analyses on the chosen gene.

### 2.6 Construction and validation for the costimulatory molecule risk model

The CMRG-related risk model was generated, and the score was estimated for each AML sample to quantify the costimulatory molecule patterns. Differentially expressed genes were subjected to univariate Cox analysis. Using an unsupervised clustering technique, AML patients were divided into three distinct CMRG-related gene subtypes (gene subtypes A, B, and C) based on the findings of the univariate Cox analysis. The chosen 242 patients with AML were then randomly categorized into two sets at a ratio of 1:1, a training AML set (*n* = 121) and a testing AML set (*n* = 121). Based on CMRG-related genes with prognostic value, we carried out the Lasso Cox algorithm to minimize the over-fitting risk in the training AML set. We next analyzed each independent variable’s change and established a risk model by using 10-fold cross-validation. Candidate genes were further analyzed and chosen in the training AML set based on the using multivariate Cox regression results. The costimulatory molecule signature was calculated using the data of each gene’s coefficient and expression. Then, based on the median score of whole samples in the AML training set, samples of two sets were respectively divided into low- or high-risk groups. Survival analysis and the creation of receiver operating characteristic (ROC) curves were performed on AML patients belonging to two risk categories.

### 2.7 Drug susceptibility analysis

To explore differences in the chemotherapeutic drug curative effect in AML patients between two risk groups from the whole set, we calculated the values of semi-inhibitory concentration (IC50) of drugs using the “pRRophetic” package in R software.

### 2.8 Statistical analyses

All statistics were analyzed in R software (version 4.1.0). Statistical significance was all set at *p* < 0.05.

## 3 Results

### 3.1 Genetic and transcriptional alterations of costimulatory molecule-related genes in patients with acute myeloid leukemia

**FIGURE 1:**
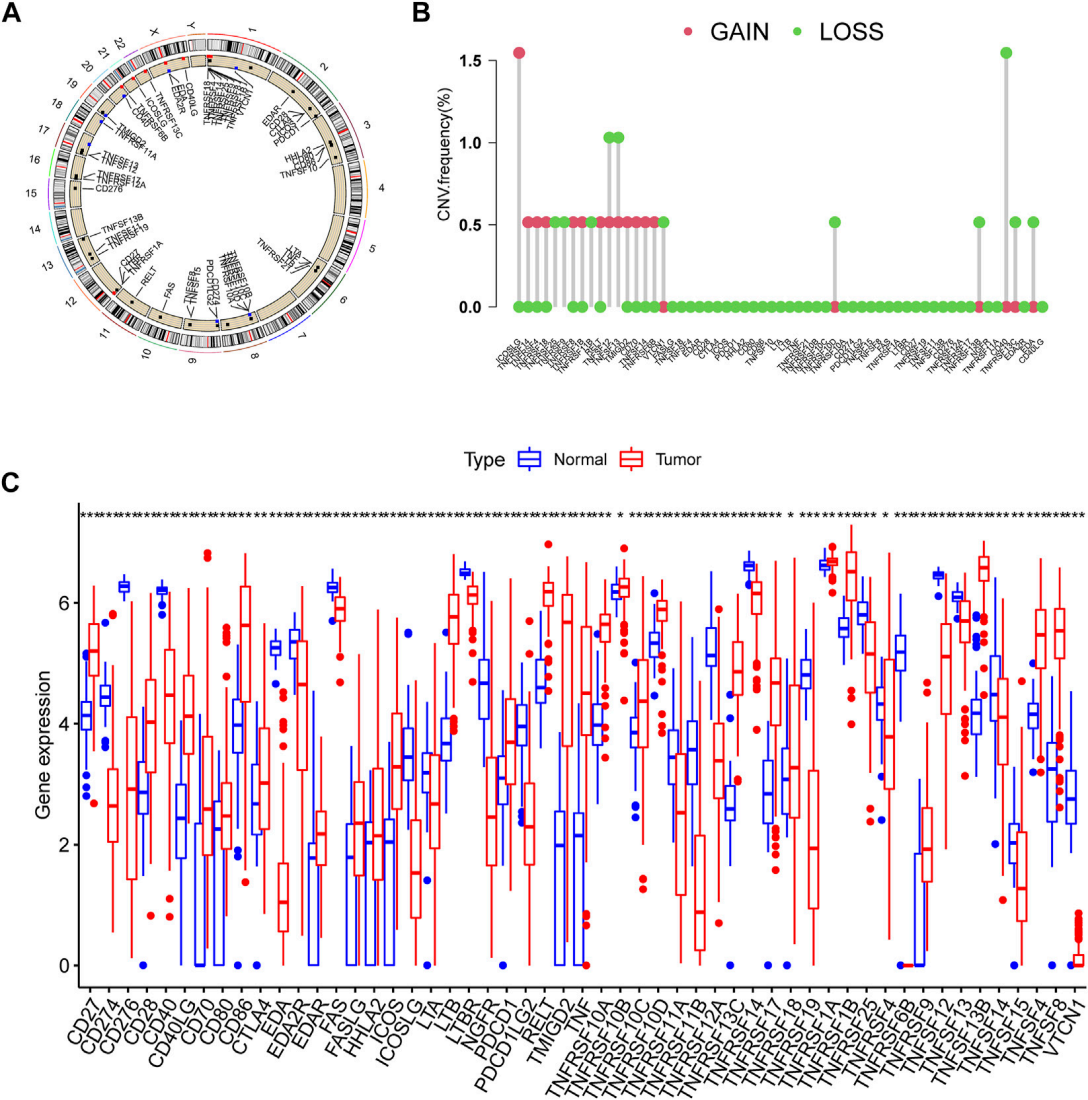
Genetic and transcriptional alterations of CMRGs in AML patients. **(A)** Locations of CNV alterations in CMRGs on 23 chromosomes. **(B)** Frequencies of CNV loss and CNV gain among CMRGs. **(C)** Expression distributions of CMRGs between normal samples from GTEx and AML samples from TCGA database.

This research contained 60 CMRGs in total. We firstly investigated the somatic copy number variations in the 60 CMRGs. Figure 1A showed the locations of the copy number variation (CNV) alterations in the CMRGs on their respective chromosomes. Among all the CMRGs, ICOSLG, TNFRSF14, TNFRSF4, TNFRSF18, TNFRSF8, TNFRSF1B, RELT, TMIGD2, CD70, TNFSF14, and TNFRSF6B had widespread CNV increases, while TNFRSF25, TNFRSF9, TNFRSF11B, VTCN1, TNFRSF10D, TNFRSF13B, CD40, TNFRSF13C, and EDA showed CNV decreases (Figure 1B). A comparison on the mRNA levels of CMRGs between AML and normal samples revealed that the majority of CMRGs were positively linked with CNV alteration. Some CMRGs with CNV gain, including TNFRSF18, TNFRSF1B, RELT, TMIGD2, and CD70, were significantly elevated in samples from the AML cohort. Meanwhile, CMRGs with CNV loss, including TNFRSF25, TNFRSF11B, VTCN1, CD40, and EDA, were expressed at lower levels in patients with AML when compared to normal samples (Figure 1C), suggesting CNV might participate in regulating the CMRGs’ expression. However, several CMRGs with a high frequency of CNV gain or loss did not vary between AML and normal samples. Thus, CNV is not the sole factor involved in CMRGs’ expression regulation. Both the genetic landscape and expression levels of CMRGs were significantly different between AML samples and controls, showing that CMRGs play a latent role in the oncogenesis of AML.

### 3.2 Identification of costimulatory molecule subtypes in acute myeloid leukemia and their tumor microenvironment characteristics

**FIGURE 2:**
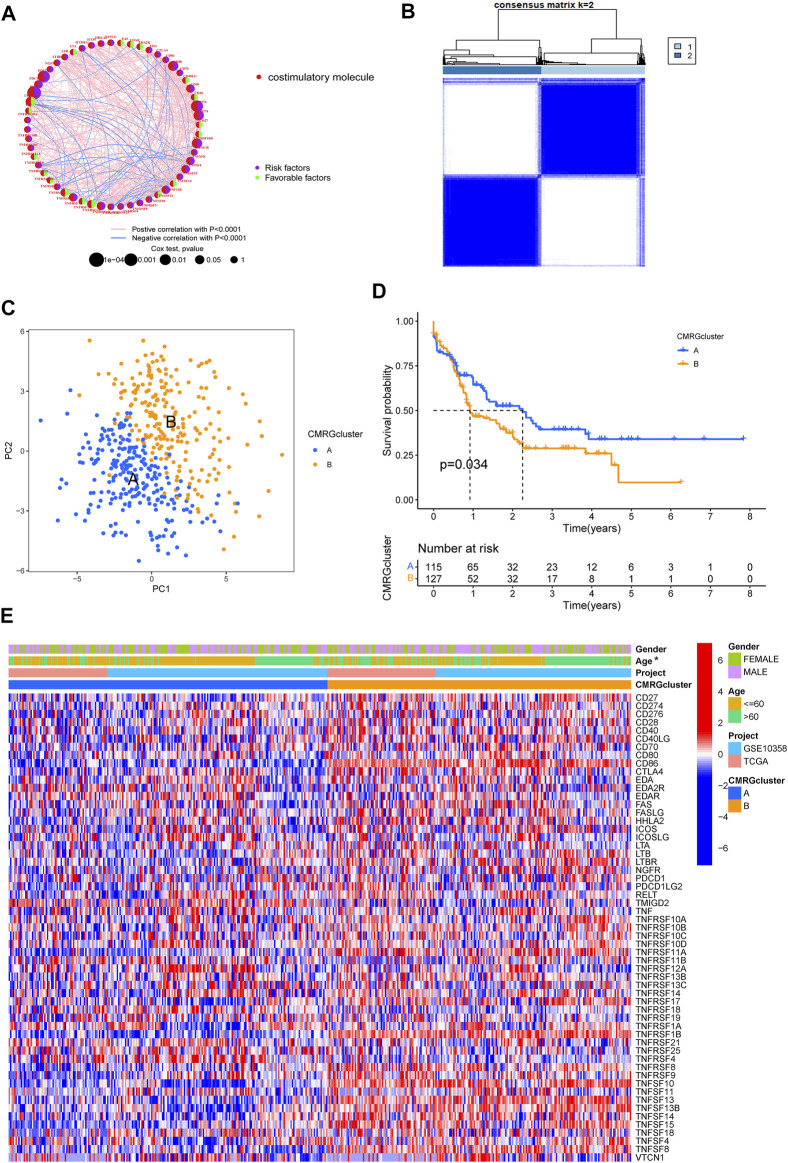
Identification of costimulatory molecule subtypes and related biological characteristics. **(A)** Interactions among 60 CMRGs in patients with AML. **(B)** Consensus matrix heatmap defining two costimulatory molecule clusters. **(C)** PCA analysis between the two costimulatory molecule subtypes. **(D)** Result of the univariate analysis of CMRGs. **(E)** Differences between the two subtypes in clinical features, as well as the CMRGs’ expression levels.

A total of 455 patients from two AML cohorts (TCGA-AML and GSE10358) were integrated into our study at the beginning. Since only 242 AML samples had information on clinical results, we extracted their data of them for further analysis. The univariate Cox regression and Kaplan-Meier analyses revealed that 44 CMRGs were found might be related to the prognosis of AML (Supplementary Figure S1). A costimulatory molecule network illustrated the full picture of CMRG connections, regulator linkages, and their predictive relevance in AML patients (Figure 2A). To further investigate the expression features of CMRGs in AML, we categorized the AML samples based on the expression profiles of 60 CMRGs using a consensus clustering technique. We found that k = 2 seemed to be the ideal choice for classifying the complete cohort into two categories (Figure 2B). Next, the results of PCA analysis revealed the obvious costimulatory molecule differences between the two costimulatory molecule subtypes (Figure 2C). Kaplan-Meier curves then showed that subtype A patients had longer overall survival than subtype B patients (Figure 2D). Furthermore, we made a comparison of the clinical features between the two various subtypes of AML. As demonstrated in Figure 2E, cluster A was more strongly associated with a younger age than cluster B.

**FIGURE 3:**
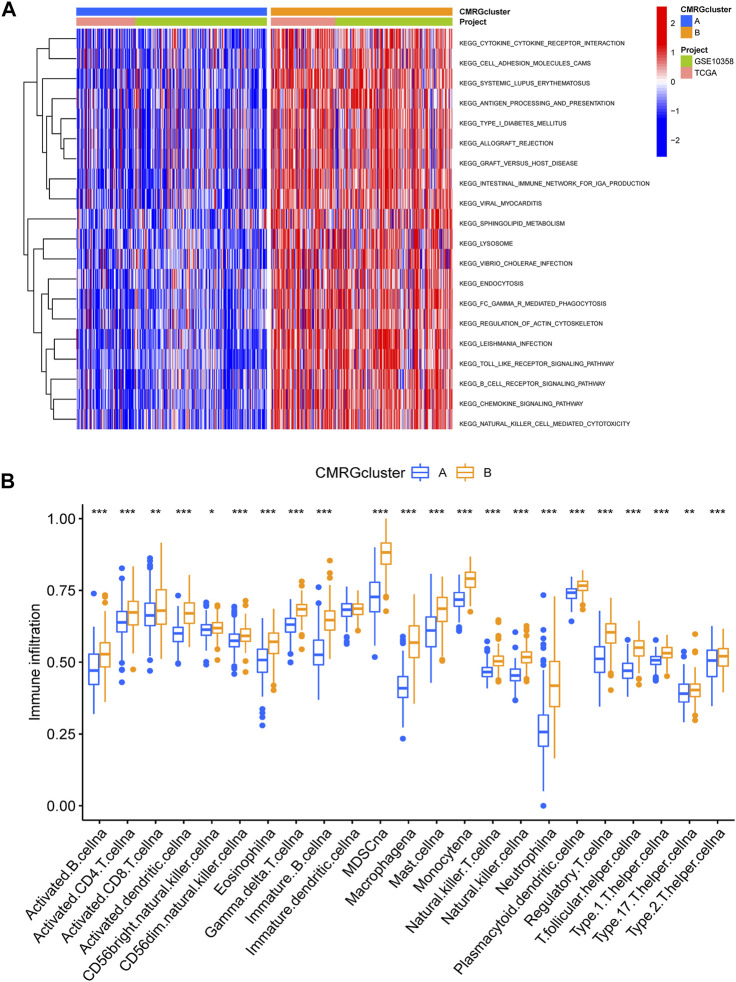
Correlations of immune cell infiltration. **(A)** GSVA analysis between two costimulatory molecule subtypes. **(B)** The abundance of 22 infiltrating immune cells.

We found that subtype B was enriched in activated immune pathways, such as cytokine receptor interaction, cell adhesion molecules, graft versus host disease, antigen processing and presentation, B cell receptor signaling pathway, allograft rejection, Toll-like receptor signaling pathway, regulation of actin cytoskeleton, and chemokine signaling pathway (Figure 3A). To investigate the functional role of CMRGs in the TME of AML, we used the CIBERSORT algorithm to analyze the correlations between the costimulatory molecule subtype and the 22 immune cell subsets of each AML sample. As shown in Figure 3B, there had been significant differences in most immune cell infiltration between subtype A and subtype B. The infiltration levels of activated CD8+ T cells, activated B cells, activated CD4+ T cells, natural killer cells, immature B cells, activated dendritic cells, monocytes, macrophages, mast cells, neutrophilia, regulatory T cells, follicular helper T cells, type 1 helper T cells, type 2 helper T cells and type 17 helper T cells were higher in patients in subtype B than in those in subtype A.

### 3.3 Gene subtypes derived from differentially expressed genes

**FIGURE 4:**
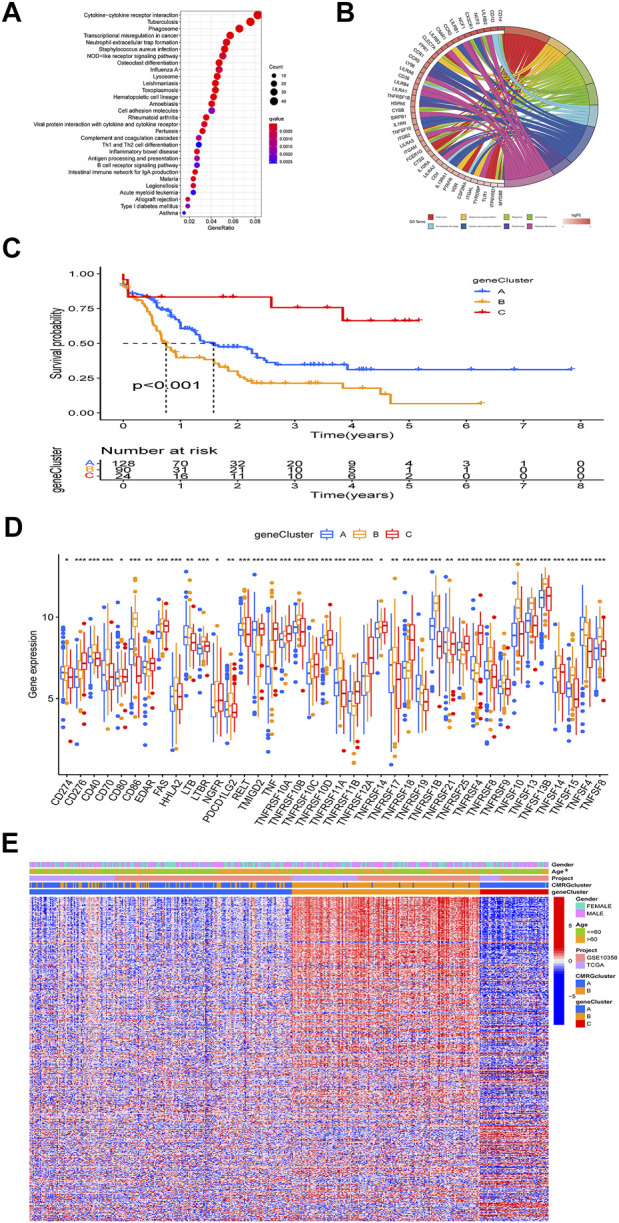
Identification of gene subtypes. **(A)** Bubble plot of KEGG enrichment analyses based on differentially expressed genes between two subtypes. **(B)** Ring plot showing results of GO enrichment. **(C)** Kaplan-Meier curve analysis for overall survival of the three gene subtypes. **(D)** Differences in the CMRGs’ expression among the three gene subtypes. **(E)** Relationships between clinical features of AML patients and gene subtypes.

The biological role of costimulatory molecule patterns in AML remains unknown. We used the “limma” package in R software to identify costimulatory molecule subtype-related differential expressed genes and then carried out the functional enrichment analysis based on them (Figures 4A,B). KEGG analysis indicated these identified costimulatory molecule subtype-related genes enriched in pathways related to cancers and immunology, suggesting that costimulatory molecules play vital roles in TME regulation (Figure 4A). The GO chord in Figure 4B also showed the GO mainly enriched terms and the significantly involved differential expressed genes. We then carried out the univariate Cox regression analysis on the subtype-related genes to screen out 501 genes that were closely related to overall survival time (Supplementary Table S2). To further explore potential regulation mechanisms, we performed a consensus clustering algorithm, which divided all AML patients into three subtypes named gene subtypes A-C. Kaplan-Meier curve in Figure 4C showed that patients in the subtype C group had the best favorable clinical outcome, whereas patients in subtype B showed the worst overall survival. The costimulatory molecule subtypes showed significant differences in the expression levels of CMRGs, which was consistent with the results of the costimulatory molecule patterns (Figure 4D). Moreover, costimulatory molecule gene subtype B patterns seemed more associated with elder age (Figure 4E).

### 3.4 Construction and validation of the costimulatory molecule-related gene-based risk model

**FIGURE 5:**
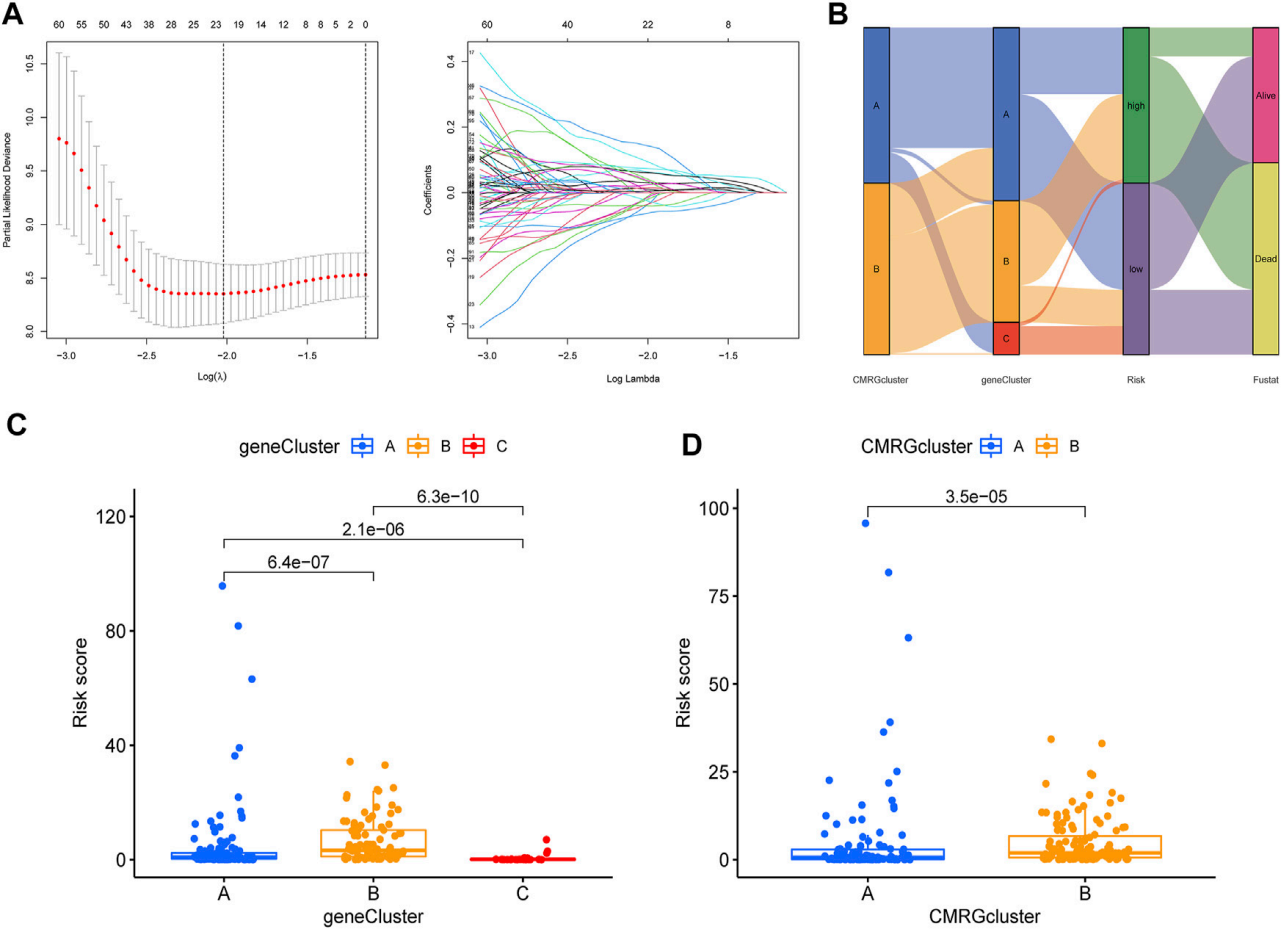
Construction of CMRG-related signature in the training set. **(A)** Results of LASSO analysis. **(B)** Alluvial diagram illustrating the subtype distributions in the training set. **(C)** Differences in risk model scores among the three gene subtypes. **(D)** Differences in risk model scores between the two costimulatory molecule subtypes.

The CMRG-related prognostic signature was then established. First, we randomly divided the AML patients into two groups, including a training group (*n* = 121) and a testing group (*n* = 121) at a ratio of 1:1. Following, LASSO and multivariate Cox analyses for costimulatory molecule subtype-related differentially expressed genes were carried out to identify CMRG-related genes with prognostic value in AML patients. According to the minimum partial probability of deviance (Snyder et al., 2014), hub overall survival-related genes remained after LASSO regression analysis (Figure 5A). We then carried out a multivariate Cox analysis to finally obtain 15 ones (GPR18, LGALS1, AOAH, DNMT3B, CBR1, ANKRD55, SIRPB2, DPY19L2, IL1R2, ST8SIA4, DOC2A, SERPINI2, GZMB, TNNT1, and SORCS2), which included nine high-risk genes and six low-risk genes (Supplementary Table S3). Hence, the CMRG-related risk model was constructed as follows: Risk score = -0.4339 * GPR18 + 0.3393 * LGALS1 - 0.3897 * AOAH + 0.3038 * DNMT3B + 0.2168 * CBR1 + 0.3566 * ANKRD55–0.3112 * SIRPB2 - 0.2369 * DPY19L2 + 0.1176 * IL1R2 -0.3160 * ST8SIA4 -0.2405 * DOC2A + 0.4186 * SERPINI2 + 0.3901 * GZMB + 0.1895 * TNNT1 + 0.3065 * SORCS2. The score of each AML patient was measured according to the signature, the median of which was chosen as the cutoff to divide AML patients into two risk score groups. The distribution of AML patients in the two costimulatory molecule subtypes, three gene subtypes, two risk score groups, and clinical outcomes were shown in Figure 5B. We then analyzed the risk scores and discovered a statistically significant difference among the three CMRG-related gene subtypes. As shown in Figure 5C, risk scores in the gene subtype B group were the highest, while the risk scores in the gene subtype C group were the lowest, which indicated that lower costimulatory molecule score might be related to immune activation. It was noteworthy that gene subtype B had a higher risk score than gene subtype A. Furthermore, Figure 5D showed the distribution of scores in two costimulatory molecule subtypes, where AML patients in the costimulatory molecule subtype B group had significantly higher scores than others in the costimulatory molecule subtype A group.

**FIGURE 6:**
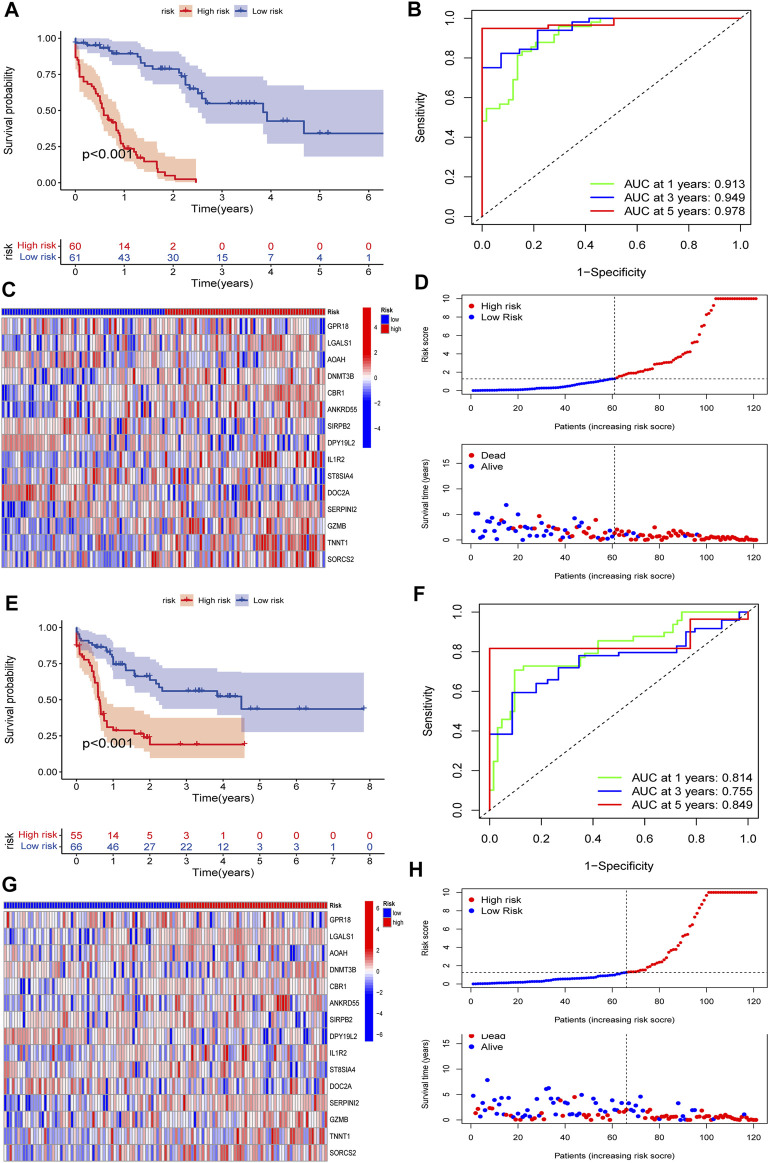
Validation of the constructed CMRG-related risk model in both training and testing sets. **(A)** Kaplan-Meier analysis of the overall survival between groups in the AML training set. **(B)** ROC curve analysis predicting the 1-, 3-, and 5-year survival sensitivity and specificity in the AML training set. **(C)** Heatmap of the 15 identified genes’ expression in the constructed risk model in the AML training set. **(D)** The CMRG-related score distribution and survival status in the AML training set. **(E)** Kaplan-Meier analysis of overall survival between groups in AML testing set. **(F)** ROC curve analysis predicting the 1-, 3-, and 5-year survival sensitivity and specificity in the AML testing set. **(G)** Heatmap showing the expression of 15 identified genes in the constructed risk model in the AML training set. **(H)** The CMRG-related score distribution and survival status in the AML testing set.

To validate the predictive value of the CMRG-related risk model in patients with AML, we separately divided AML patients in each set into two different risk groups according to the cutoff, which was the median value of those in the AML training set. In detail, AML patients whose scores were larger than the chosen cutoff were grouped as low risk, while others whose scores were smaller than the chosen cutoff were grouped as high risk. The survival curves revealed that AML patients in the training set with lower scores significantly had a favorable overall survival time when they were compared to those with higher scores (Figure 6A). In the training set, the 1-, 3-, and 5-year survival rates for AML patients were respectively 0.913, 0.94, and 0.978, which were represented by AUC values (Figure 6B). The heatmap showed the 15 CMRG-related genes’ expression in two risk groups in the AML training set (Figure 6C). The distribution of the costimulatory molecule risk score revealed that the overall survival time of AML patients in the training set decreased with the scores increase (Figure 6D). Patients in the AML testing set were similarly divided into two risk categories using the same algorithm and cutoff as the training set. Survival analysis revealed that the group with lower scores had considerably better clinical results (Figure 6E). While studies of 1-, 3-, and 5-year prognosis prediction efficiencies revealed that the CMRG-related risk score maintained high AUC values, this was not the case for the 5-year prognostic efficiency (Figure 6F). The heatmap and survival status of AML patients showing the variation tendencies of two risk groups from the testing set were shown in Figures 6G,H, respectively, further confirming the costimulatory molecule signature had an excellent ability to predict the clinical outcome of patients with AML.

### 3.5 Evaluation of tumor microenvironment and checkpoints

**FIGURE 7:**
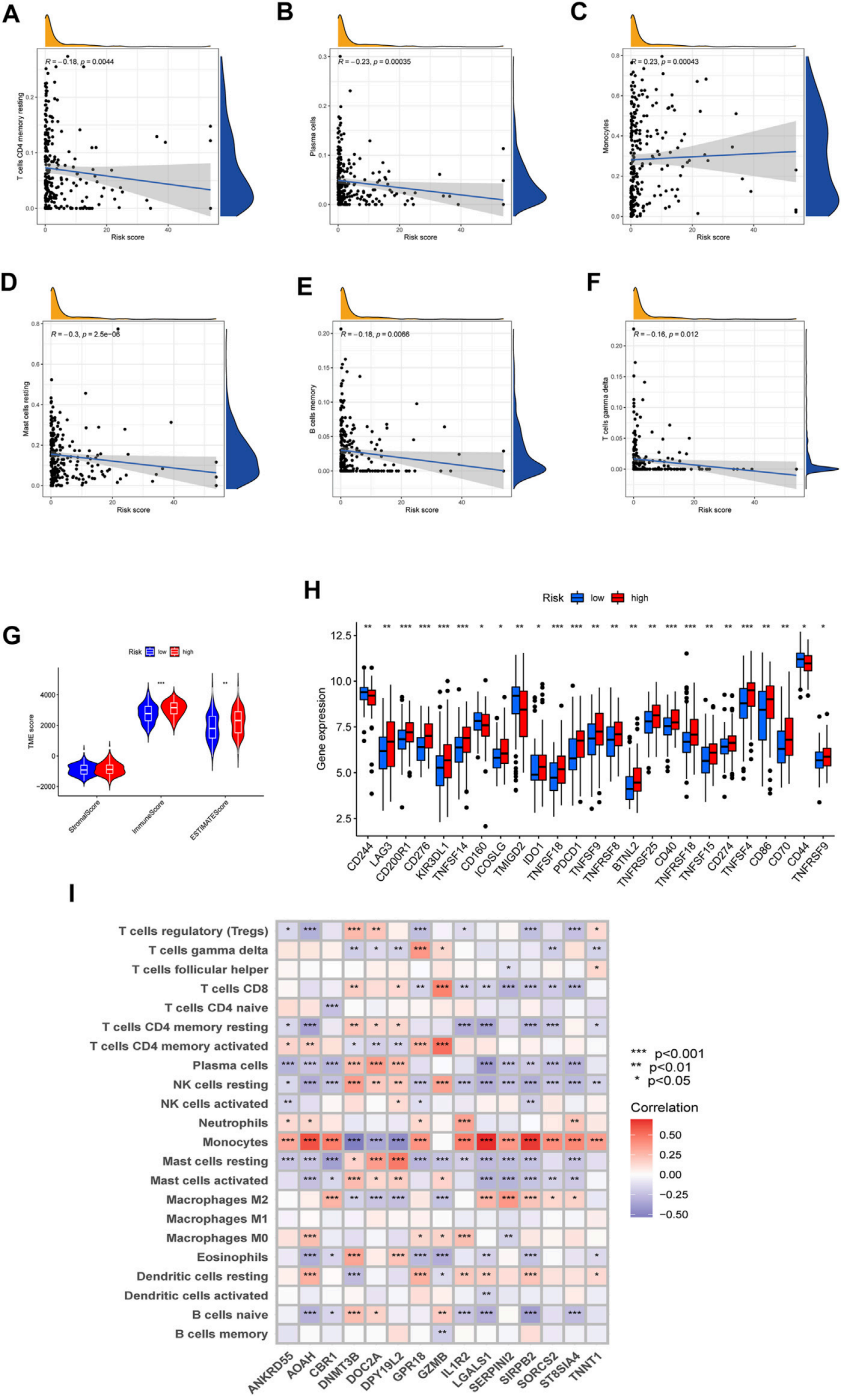
Evaluation of the TME and checkpoints. **(A–F)** Correlations between the risk model and immune cell types. **(G)** Correlations between the CMRG signature and immune or estimate scores. **(H)** Expression of immune checkpoints in groups. **(I)** Correlations between the 15 identified genes in the proposed risk model and immune cell abundance.

To investigate the difference in TME and expression of checkpoints between two risk groups divided by constructed signature, we divided all the 242 AML patients into two groups according to cutoff and perform the CIBERSORT algorithm, by which we could assess the correlations between risk scores and immune cell abundance. As the scatter diagrams in Figure 7A showed the constructed costimulatory molecule risk score was positively correlated with monocytes and was negatively correlated with plasma cells, resting memory CD4+ T cells, resting mast cells, memory B cells, and gamma delta T cells (Figures 7A–F). Higher risk scores were also closely linked with higher immune scores as well as higher estimate scores (Figure 7G). Figure 7H shows that a total of 25 immune checkpoints, including LAG3, IDO1, and PDCD1, were different between the two groups from the total AML set. Moreover, the majority of immune cells were linked with the genes in the proposed model (Figure 7I).

### 3.6 Analysis of drug susceptibility and validation in pediatric acute myeloid leukemia

We then selected chemotherapy drugs to evaluate the treatment sensitivities of patients in different risk AML groups. Interestingly, we found that the patients with higher costimulatory molecule scores had lower IC50 values for WZ-1-84, WO2009093972, SL 0101–1, S-trityl-L-cysteine, Roscovitine, Rapamycin, Parthenolide, NVP-TAE684, Kin001-135, GNF-2, Dasatinib, CGP-082996, CGP-60474, Bortezomib, and AZ628, while IC50 values of chemotherapeutics such as VX-702, Vorinostat, Thapsigargin, SB 216763, Plx4720, PF-562271, OSI-906, MK-2206, Mitomycin-C, Midostaurin, Gemcitabine, Embelin, Cytarabine, BX-795, Bosutinib, BI-D1870, AZD7762, Axitinib, AP-24534, AKT inhibitor III, AG014699 and ABT263 were significantly lower in the patients with lower costimulatory molecule scores (Supplementary Figure S2). Moreover, to validate the prognostic performance of the costimulatory molecule signature in pediatric AML, we extracted the data of samples from the TARGET database. It is obvious that most CMRG-related genes in the proposed signature were differentially expressed between pediatric AML samples and control samples (Supplementary Figure S3), suggesting these CMRG-related genes also played important roles in pediatric AML.

## 4 Discussion

The involvement of costimulatory molecule signal in innate immunity and anticancer effects has been shown by research (Tang et al., 2018; Zhang et al., 2020). Nonetheless, the global impact mediated by the combined numerous CMRGs has not been completely understood. The current investigation indicated widespread alterations in the CMRGs at the levels of transcription and genetics (Figure 1). We next identified two unique costimulatory molecule subtypes according to the expression levels of 60 CMRGs. Patients in the subtype B group showed more advanced clinical characteristics and worse overall survival rates (Figure 2). The TME features between subtypes also varied. The costimulatory molecule subtypes were distinguished by the activation of the immune system (Figure 3). The differences in transcriptomes of mRNA between costimulatory molecule subtypes were strongly associated with immune biological pathways (Figure 4). Following, we identified three gene subtypes according to the expression of differentially expressed genes between two costimulatory molecule subtypes. We finally construct the effective prognostic costimulatory molecule signature and validated its predictive ability (Figures 5,6). Diverse costimulatory molecule scores were associated with markedly different prognosis, clinical features, immunological checkpoints, TME, and drug susceptibility among AML patients (Figure 7 and Supplementary Figure S2). Further validation revealed that the identified CMRGs in the proposed signature might also play important roles in pediatric AML (Supplementary Figure S3). The prognostic model may be used to predict the prognosis of AML patients and will aid in the comprehension of AML’s molecular process.

The clinical outcome of AML after conventional chemotherapy is still poor (Prebet et al., 2012; Boddu et al., 2017). Despite the achieved advances in immunotherapy in recent years, the outcomes of AML patients are heterogeneous (Johnson et al., 2022), which highlights the role of TME (Huang et al., 2019). Immune cells, the main components of TME, take part in various immune activities (Seager et al., 2017). Evidence has shown the effects of TME on the development, progression of tumors, as well as therapeutic resistance (Hinshaw and Shevde, 2019; Banerjee et al., 2021). In our study, the costimulatory molecule pattern which was characterized by immune inhibition was found significantly associated with higher costimulatory molecule scores. Also, the characteristics of TME in AML, as well as the 22 immune cell abundance, differed significantly between costimulatory molecule subtypes (Figure 3). More and more evidence has shown that immune cells play vital roles in AML (Ma Y et al., 2020). Macrophages could support the progression and tumor drug resistance by providing nutritional support (Vitale et al., 2019; Boutilier and Elsawa, 2021). They were also reported as pro-tumoral, as well as neutrophils, both of which promote invasion and metastasis and suppress surveillance (Powell and Huttenlocher, 2016; Hinshaw and Shevde, 2019; Wu et al., 2020). Dendritic cells are tumor-promoting (Chaput et al., 2008; Veglia and Gabrilovich, 2017; Wculek et al., 2020), while natural killer cells eliminate tumor cells. Additionally, regulatory T cells also suppress immunological responses with anti-cancer impact (De Simone et al., 2016; Elkord and Sasidharan, 2018). Gamma delta T cells may efficiently identify and eliminate tumor cells, hence they could suppress the progression of tumors (Ma R et al., 2020). Subtype B and high costimulatory molecule scores, with worse prognosis, had higher activated CD4+, CD8+, and gamma delta T cell infiltration, which suggest that they play negative roles in the development of AML.

Checkpoints in the immune system play crucial roles in the immunosuppression of most cancers (Pardoll, 2012; Ribas and Wolchok, 2018). In hematological malignancies, common immune checkpoint targets are reported mainly include PDCD1, IDO1, PD-L1, LAG3, and CTLA-4 (Ok and Young, 2017). Here, we found three common immune checkpoints, including PDCD1, LAG3, and IDO1, elevated in the group with higher costimulatory molecule scores (Figure 7), indicating the state of immunosuppression in the bone marrow microenvironment. While the immunosuppressive state of AML patients was reported might be the reason causing the immunotherapy resistance (Xu et al., 2021). In addition, tumor cells in leukemias could help create the immunosuppressive state by generating energy that is enough for escaping from antitumor immune surveillance (Xu et al., 2021). Finally, we explored the association between the signature and medication response to facilitate the development of individualized treatment plans. It is crucial to identify novel biomarkers for immunotherapy patient selection. The findings indicated that low-risk individuals may benefit from these medications. Our signature may further assist in identifying patients who may benefit from antitumor immunotherapy and aid in the formulation of a more rational and effective treatment regimen, therefore contributing to personalized therapy for individuals with varying risk profiles.

Absolutely, this study had several limitations. First of all, the analyses were all carried out on the samples obtained from the public databases with retrospective data. Large-scale prospective studies *in vivo* and *in vitro* need to be performed to confirm the findings in this study. Also, the number of clinical characteristics that both datasets in this study contained is too small. Some common and crucial clinical variables for AML patients were unavailable for further analysis, which may affect the confirmation of the prognostic value of constructed signature in the immune response in AML.

## 5 Conclusion

Our analysis based on the CMRGs revealed a potential regulatory mechanism in AML, by which they might affect the TME, clinical features, drug susceptibility, and prognosis of patients with AML. These findings highlight the applications of CMRGs in AML in clinics and provide ideas for applying personalized immunotherapy to AML patients.

## Data Availability

The datasets presented in this study can be found in online repositories. The names of the repository/repositories and accession number(s) can be found in the article/Supplementary Material.

## Supplementary material

Supplementary Figure S1 The Kaplan-Meier survival plots of CMRGs in the AML patients.

Supplementary Figure S2 Relationships between the CMRG-related signature and chemotherapeutic sensitivity.

Supplementary Figure S3 Expression of the 15 identified genes in the constructed risk model in pediatric AML.

Supplementary Table S1 The details of 60 costimulatory molecule-related genes (CMRGs).

Supplementary Table S2 Results of the univariate regression analysis in AML patients based on the CMRG-related differentially expressed genes.

Supplementary Table S3 Results of the multivariate Cox regression analysis based on the CMRG-related genes with prognostic value in patients with AML.
