# Supplementary figures and images for: Identification of a prognostic model based on costimulatory molecule-related subtypes and characterization of tumor microenvironment infiltration in acute myeloid leukemia
Source: Front Genet. 2022 Aug 19;13:973319. doi: 10.3389/fgene.2022.973319 (PMC9437340; doi:10.3389/fgene.2022.973319)

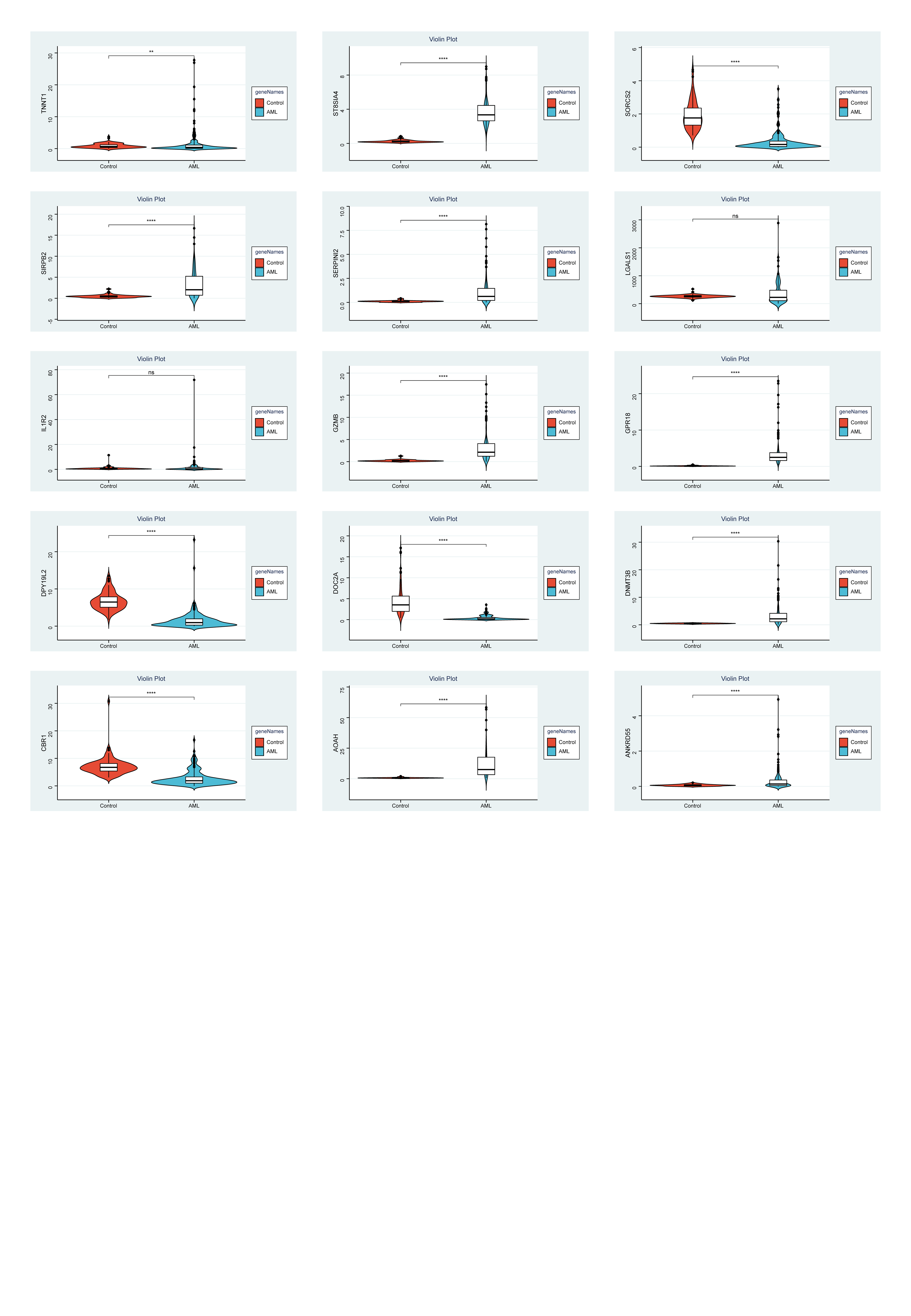

Supplement: Supplementary file 3 [file Image3.TIF]

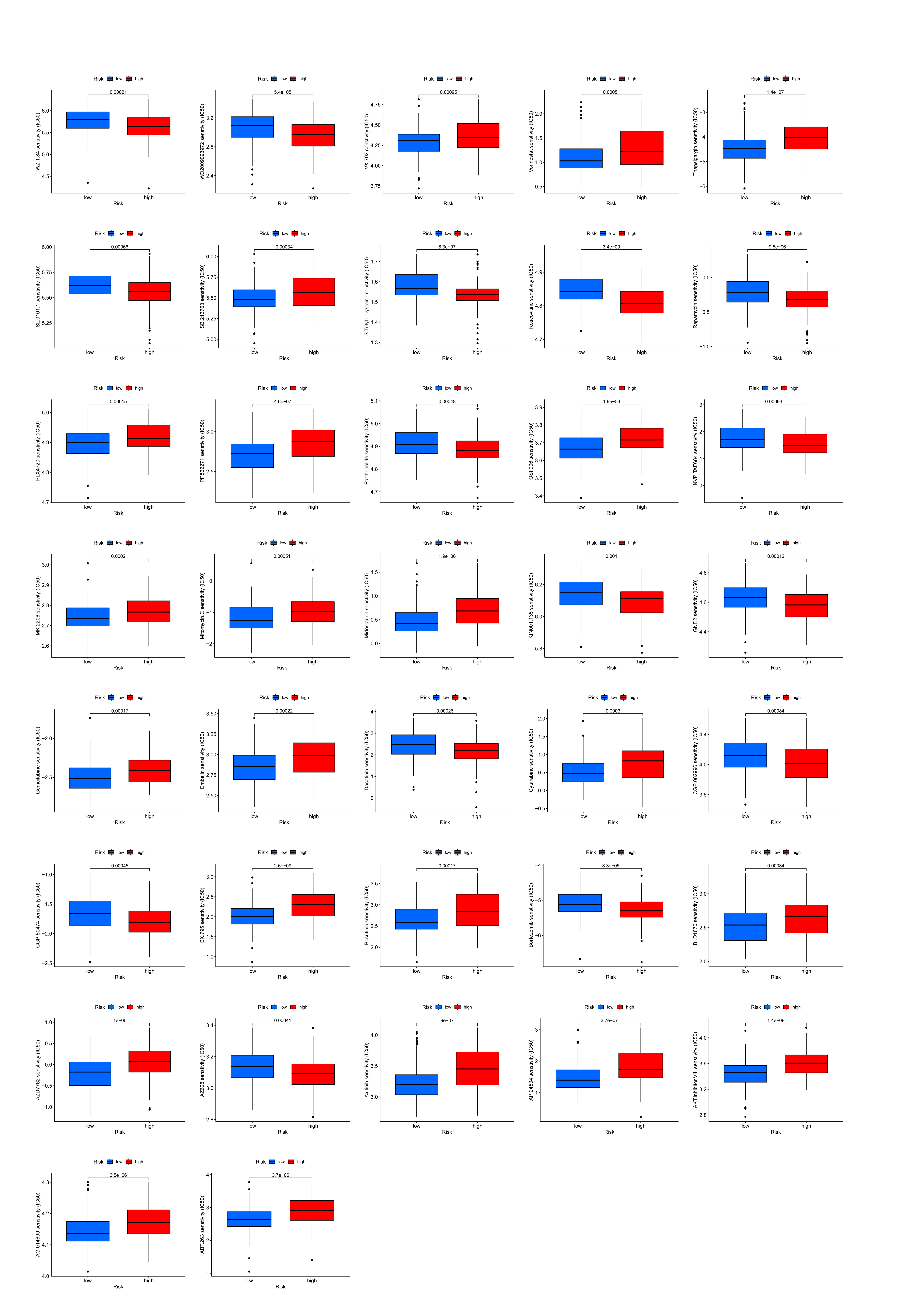

Supplement: Supplementary file 4 [file Image2.TIF]

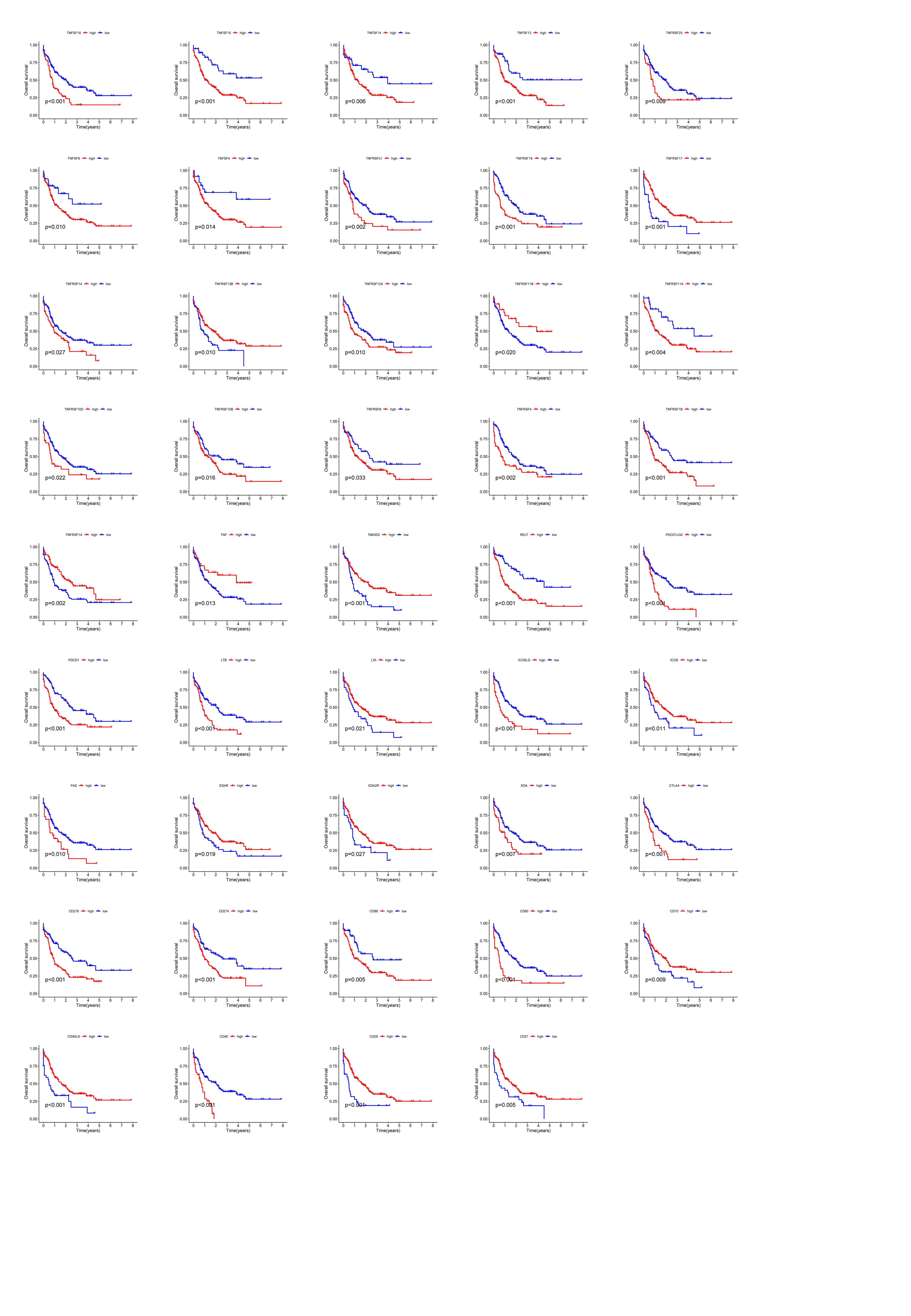

Supplement: Supplementary file 5 [file Image1.TIF]
